# Laser therapy versus pulsed electromagnetic field therapy as treatment modalities for early knee osteoarthritis: a randomized controlled trial

**DOI:** 10.1186/s12877-022-03568-5

**Published:** 2023-03-16

**Authors:** Michal Elboim-Gabyzon, Fouad Nahhas

**Affiliations:** Physical Therapy Department, Faculty of Social Welfare and Health Sciences, University of Haifa, 188 Hushi Abba Boulevard, 3498837 Haifa, Israel

**Keywords:** Pulsed electromagnetic field therapy, Low-level laser therapy, Knee osteoarthritis, Pain, Function

## Abstract

**Background:**

This randomized controlled trial aimed to compare the effects of pulsed electromagnetic field therapy (PEMFT) and low-level laser therapy (LLLT) on pain and physical function of participants with knee osteoarthritis (KOA).

**Methods:**

According to the Kellgren–Lawrence classification, participants with grade 2–3 KOA were randomized to receive PEMFT or LLLT for six sessions lasting 15 min/session over a 3-week period. Pain at rest and when walking, standing from a sitting position, and climbing the stairs was assessed using the visual analog scale. Functional level was measured by the Western Ontario and McMaster Universities Osteoarthritis Index (WOMAC), timed up-and-go test (TUG), and 10-m walk (10 MW) test. Measurements were obtained before and after the interventions. Significance was determined at *p* ≤ 0.05.

**Results:**

Forty participants were included in the study. Pain and physical function improved significantly (*p* < 0.0001) in both groups. PEMFT was significantly more effective in reducing pain at rest, when standing from a sitting position, and when climbing the stairs, and in improving both WOMAC scores and TUG results (*p* ≤ 0.0003). The improvements in pain during the activities and the WOMAC scores reached the minimal clinically important difference. No adverse events occurred.

**Conclusion:**

Six sessions of PEMFT and LLLT had immediate positive effects on pain and physical function in individuals with low-grade KOA, with PEMFT resulting in significantly better results.

**Trial registration:**

ISRCTN registry trial ID: ISRCTN17001174

**Supplementary Information:**

The online version contains supplementary material available at 10.1186/s12877-022-03568-5.

## Background

Knee osteoarthritis (KOA) is the most common chronic joint disorder worldwide and is the most frequent cause of activity limitation and disability in elderly adults [1, 2]. Additionally, KOA causes a significant burden on the global health care system and workforce [1, 2]. Thus, KOA is a condition that requires significant research and clinical attention, which is further reinforced by the expectation that the global incidence of KOA will increase due to the increasing prevalence of the main risk factors of KOA, including aging and obesity [1, 2].

The diagnosis of KOA is based on clinical symptoms, including knee pain that worsens with walking and climbing the stairs, limited range of motion at the knee, and weakness of the knee muscles. These symptoms are inimical to daily functioning that impairs the patient’s quality of life [3]. KOA is also associated with an increased incidence of falls [4]. The severity of the disease is determined based on the degree of clinical symptoms (intensity of pain and functional limitations) and joint damage on radiography [5]. Treatment of KOA primarily consists of symptomatic relief as disease-modifying treatments are unavailable [1]. Clinical practice guidelines are based on the disease’s severity, and treatment is divided into non-pharmacological, pharmacological, and surgical interventions [1, 6, 7]. The various treatment guidelines outline a sequential approach based on the severity of symptoms in terms of pain intensity, level of disability, and quality of life [1].

As determined by the leading authorities regarding KOA (The European Society for Clinical and Economic Aspects of Osteoporosis, Osteoarthritis, and Musculoskeletal Diseases and the Osteoarthritis Research Society International), the core KOA treatment sequence begins with patient education, physical therapy, and weight loss [8–13]. The next step entails the use of pharmacological agents for analgesia such as oral NSAIDs and intra-articular corticosteroid injections [1]. However, long term use of analgesics is limited due to adverse side effects, including possible addiction [14].

Total knee replacement (TKR) surgery is the treatment of choice in cases wherein non-surgical treatment modalities are no longer effective [15]. Despite the beneficial outcomes of TKR in improving symptoms, approximately 20% of patients who undergo TKR report dissatisfaction and experience chronic pain [16, 17]. Additionally, TKR cannot be performed in the presence of certain comorbidities, such as active knee sepsis and severe untreated or untreatable peripheral arterial disease [18]. Therefore, there is a tendency among clinical practitioners and researchers to use alternative, non-pharmacological, and less invasive modalities that have minimal side effects. These include oxygen–ozone (O_2_O_3_) therapy [19], ultrasound-guided radiofrequency ablation of genicular nerves combined with exercise therapy [20], low-level laser therapy (LLLT) [21], and pulsed electromagnetic field (PEMFT) [22]. Recently, the popularity of LLLT and PEMFT has increased, with both modalities demonstrating great potential as effective treatments for KOA [21–24]. LLLT, also termed photo-biomodulation, entails the application of non-invasive, monochromatic, collimated, and coherent light at an output power of less than 0.5 watts (defined as Class III in accordance with the FDA guidelines) [25]. LLLT does not result in perceivable heating of tissue due to its low power output; hence, it is also called “cold laser” therapy [26]. When treating musculoskeletal disorders, the wavelengths used during LLLT commonly range between 632 and 904 nm [26].

The mechanism of action of LLLT is photochemical (photobiomodulation) [26]. Light induces biochemical changes within cells, including cellular oxygenation, release of neurotransmitters associated with pain modulation (e.g., serotonin), and release of anti-inflammatory mediators [26–31]. Accordingly, LLLT may help control inflammation of the synovial membrane (synovitis) in KOA, thereby reducing the severity of symptoms [29].

Under proper irradiance and adequate irradiation time, systematic reviews reported positive effects when laser therapy was applied to cartilage defects in animal models of KOA [32]. LLLT was also found to regulate catabolic and anabolic factors in the cartilage of rabbits with progressive OA, suggesting that LLLT may prevent cartilage degradation and synovitis [33]. Recent systematic/meta-analysis/reviews [21, 34–36] advocate LLLT as a safe and effective treatment modality to reduce pain as well as to improve physical function of patients with KOA. However, critics of LLLT cite discrepancies between various studies regarding its effectiveness, with some studies reporting no beneficial effects from LLLT [28, 29, 37]. Additionally, there is a lack of information regarding the optimal treatment parameters and the dose–response relationship in patients with KOA [29, 35], which may be attributed to the heterogeneity of factors related to LLLT, such as the applied dose in terms of energy density, frequency, wavelength, treatment duration, number of treatment sessions, irradiation area, standalone vs. adjunctive therapy, or even factors related to the treated condition such as KOA severity [35].

PEMFT is a non-invasive therapeutic modality that combines magnetic electric fields generated by a pulsed electromagnetic field signal generator and a coil assembly with a two-coil array [22], with the treatment area being positioned between the two coils [22]. In clinical settings, a frequency of less than 100 Hz and a magnetic flux density between 0.1 and 30 mT are used during PEMFT [22, 38].

The rationale behind using PEMFT as a treatment modality for KOA is based on its effect on structures comprising the knee joint, including cartilage [39]. PEMFT increased bone and cartilage turnover in an animal model of osteoarthritis [40]. Additionally, it was proposed that PEMFT can regenerate cartilage by increasing the degree of proliferation as well as the degree of maturation of chondrocytes via the secretion of extracellular matrix. These processes occur through the release of anabolic morphogens, such as bone morphogenetic proteins, both in vitro and in vivo. This counteracts the progressive loss of tissue in degenerative disorders by preventing subchondral bone destruction and promoting the repair of cartilage and microarchitecture of the subchondral trabecular bone [24, 40–42]. Furthermore, in vivo studies demonstrated that PEMFT enhances the expression of adenosine receptors in chondrocytes and synoviocytes, thus modulating nociception and inflammatory processes [24, 39, 43, 44].

Over the last decade, several systematic reviews of clinical trials on the therapeutic effects of PEMFT in KOA have been conducted. However, results regarding joint pain, stiffness, and physical function post-therapy remain controversial [39, 42, 45–49]. There is a general consensus that the reason for the contradictory evidence regarding the effectiveness of PEMFT is that some studies combined other types of electromagnetic therapy (such as pulsed short-wave therapy) with PEMFT, whereas other studies simultaneously focused on both hip and knee OA; these may have resulted in biases due to the different etiologies and pathophysiology of KOA in these joints. Other criticisms of previous studies include the absence of standardized treatment protocols or explanations regarding the rationale for parameter selection [22, 24, 40, 47]. However, there is also evidence from systemic reviews that supports PEMFT in its ability in reducing knee pain [39, 42, 44, 45, 48–50] and improving physical function. Additionally, the effects of PEMFT were particularly observed on low-grade KOA and in participants younger than 65 years old [24, 51].

In a previous study [52] that compared LLLT and PEMFT in terms of their effects on bone mineral density in older adults with primary osteoporosis, both modalities were effective; however, PEMFT was superior to LLLT. However, no study has compared the effectiveness of these two modalities on KOA. Thus, the aim of this study was to compare the effects of PEMFT and LLLT on pain and physical function of patients with low-grade primary KOA.

## Methods

### Trial design and participants

This was a single-blinded (assessor), randomized, controlled clinical trial that randomized participants to receive either PEMFT or LLLT. The study was approved by the ethical review board at the University of Haifa. All participants provided written informed consent before participation and after a detailed explanation of the study objectives and design. The study was registered at the ISRCTN registry in 20/04/2022 with trial ID: ISRCTN17001174.

Participants were recruited at an outpatient orthopedic clinic in Israel. The sample size was calculated using a G power computer program based on previously reported data [49, 52]. We hypothesized that PEMFT and LLLT would result in a 20 and 13% decrease (standard deviation [SD], ± 2 points) in the WOMAC score, respectively. Assuming that a two-tailed t-test of the two independent variables (two groups) would be at a significance level of 0.05 with a statistical power of 80%, 17 participants would be required in each group. Additionally, we estimated a 15% attrition rate; therefore, 20 participants would be required in each group.

Participants were enrolled into the study if they met the following inclusion criteria: diagnosed with primary KOA, aged between 50 and 75 years, symptomatic knee pain of 6 months or longer, pain level ≥ 4 out of 10 according to the visual analog scale (VAS), independent walking ability of at least 30 m, and grade 2–3 KOA according to the Kellgren–Lawrence (KL) classification scale [53]. The KL scale consists of the following five grades: grade 0 represents a normal joint without injury; grade 1 represents onset of osteophyte formation; grade 2 represents onset of subchondral sclerosis with slight narrowing of the joint space; grade 3 represents > 50% narrowing of the joint space with osteophyte formation and high-grade subchondral sclerosis; and grade 4 represents severe stenosis accompanied by destruction of the joint [54].

The severity of KOA according to the KL scale was assessed by the referring orthopedist based on a radiographic image of the knee taken with the patient in the standing position.

Participants with secondary KOA, significant sensory disturbances in the lower extremities, uncontrolled diabetes or heart disease, body mass index (BMI) of > 40, presence of a pacemaker, previous history of lower limb surgery, implants in the body, or inability to understand simple instructions were excluded from the study.

### Randomization and interventions

Participants were randomly assigned to one of two intervention groups using a computer-generated random allocation software sequence. This task was performed by the clinic secretary, who was not involved in the study, prior to the pre-intervention assessment once the eligibility criteria were confirmed.

PEMFT and LLLT were administered by experienced physiotherapists who underwent a 1-h training of the study protocol. Although physiotherapists could not be blinded to the treatment group, they were not involved in the assessment process and were unaware of the participants’ assessment results during the study period.

Both PEMFT and LLLT were administered using a PhysioGo™ 500I device (Astar Company, Poland) with different applicators. The treatment parameters and intensity of the modalities were set as per the fixed protocol of the device that was appropriate for KOA and according to the patient’s group allocation. The intervention was performed with the patient in the supine position with the headrest raised and their knee supported on a pillow, resulting in approximately 15°–30° of flexion depending on the comfort and pain reported by the patient. Each treatment modality was administered for six sessions over a 3-week period. Participants were instructed to continue with their usual daily activities during the study period without starting any new physical activity that might aggravate their pain.

LLLT was performed while wearing goggles, and the treatment parameters were as follows: power, 100%; dose, 8 J/cm^2^; frequency, 2 Hz; duty factor, 75%; and treatment area, 20 cm^2^ applied over five points over the anterior part of the articular space for 3 min at each point for a total time of 15 min (see Additional file 1). The instruction given to the patients was that no sensation or a sensation of low-level warmth might be experienced in the irradiated area, which would resolve immediately after treatment.

For PEMFT, the applicator was focused over the medial and lateral sides of the knee (see Additional file 1). The parameters were as follows: rectangular field shape; frequency, 30 Hz; intensity, 10 mT; and treatment time, 15 min. The instruction given to the patients was that no sensation would be perceived.

### Outcomes

Two assessments were performed: the first was performed prior to treatment group allocation, and the second was performed at the end of the treatment sessions. All assessments were performed by the same trained physiotherapist who was blinded to the treatment group allocation and was not involved in the interventions. The primary outcomes were pain intensity and functional level.

#### Pain intensity

Pain intensity at rest, when walking, when standing from a sitting position, and when climbing the stairs were assessed using the visual analogue scale (VAS), which is an assessment tool composed of a straight horizontal line of fixed length (usually 100 mm). The ends of the line represent the extreme limits of pain, with 0 indicating no pain and 10 representing extreme pain. The VAS was found to have excellent test–retest reliability (intraclass correlation coefficient = 0.97) in participants with KOA [55].

#### Functional level

The functional level was assessed using the Hebrew version of the Western Ontario and McMaster Universities Osteoarthritis Index (WOMAC) and the following two performance tests: A) timed up-and-go (TUG) test and B) 10-m walk (10 MW) test.

The WOMAC is a self-reported disease-specific questionnaire that specifically assesses pain intensity, stiffness, and level of physical functioning in participants with KOA or hip osteoarthritis [56]. The questionnaire is composed of 24 items divided into three subscales: 1) pain, 5 items; stiffness, 2 items; and physical functioning, 17 items. The total score ranges from 0 to 96, with higher scores indicating higher pain intensity, elevated levels of stiffness, and more functional limitations [57]. WOMAC is frequently used in clinical and research settings and has been translated into many languages because of its ease of use and good psychometric properties [58]. The Hebrew version was found to be a valid and reliable tool to assess KOA severity using Pearson’s correlation test [59]. Additionally, a significant correlation (*p* < 0.01) was demonstrated between the Hebrew version of the WOMAC and VAS scores for pain and dysfunction [53].

The TUG test assesses functional mobility by measuring the time required to stand from a chair, walk 3 m, turn, return to the chair, and sit [60]; higher scores reflect worse functional mobility [60]. The TUG test was recommended by the Osteoarthritis Research Society International as one out of the five essential performance-based tests that should be included in the assessment of individuals diagnosed with KOA. The TUG test assesses the performance of activities that are usually impeded by KOA and demonstrates good psychometric properties [61]. Additionally, the TUG test has excellent reliability in participants with grades 1–3 KOA, with an intra-rater and inter-rater reliability of 0.97 and 0.96, respectively [62].

The 10 MW test evaluates walking ability by measuring the time (in seconds) required to walk 10 m [63]. A subject is asked to walk a 14-m indoor course that is delineated with tape markers at 0, 2, 12, and 14 m. The actual course length is 10 m (between the 2- and 12-m marks) to minimize the effects of acceleration and deceleration [64]. The 10 MW test is frequently used to assess walking ability among people with KOA [64, 65] as its reliability and validation for various conditions and in diverse populations have been established [66, 67].

Secondary outcome measures were the number of participants that dropped out in each group and the occurrence of adverse events.

### Statistical analysis

Descriptive statistical analysis included means and SD for continuous variables, and numbers and percentages for categorical variables. As the distribution of the results was non-normal, non-parametric comparison tests were used. Pre-intervention comparisons of baseline characteristics were performed using the Chi-square test for sex, KL grade, and involved side. The Wilcoxon two-sample test was used to analyze age, BMI, and pain onset. Changes in treatment effectiveness are presented as the difference between the post-intervention and baseline values (termed delta). The time effect of the PEMFT and LLLT groups was analyzed separately using Friedman’s Chi-square test along with analysis of the effect size by using the Friedman test effect size. The magnitude of the effect size was defined by Kendall’s W value as 0.1–0.3, small effect; 0.3–0.5, moderate effect; and ≥ 0.5, large effect. The group effect was analyzed using the Wilcoxon two-sample t test along with the Wilcoxon effect size test (r). The magnitude of the effect was defined as 0.10–< 0.3 for a small effect, 0.30–< 0.5 for a moderate effect, and ≥ 0.5 for a large effect. The group × time effect was analyzed using Wilcoxon two-sample T to explore whether the time effect was different between the two groups. The Wilcoxon effect size test (r) was used to assess the effect size. Statistical significance was set at *p* < 0.05.

## Results

**Fig. 1:**
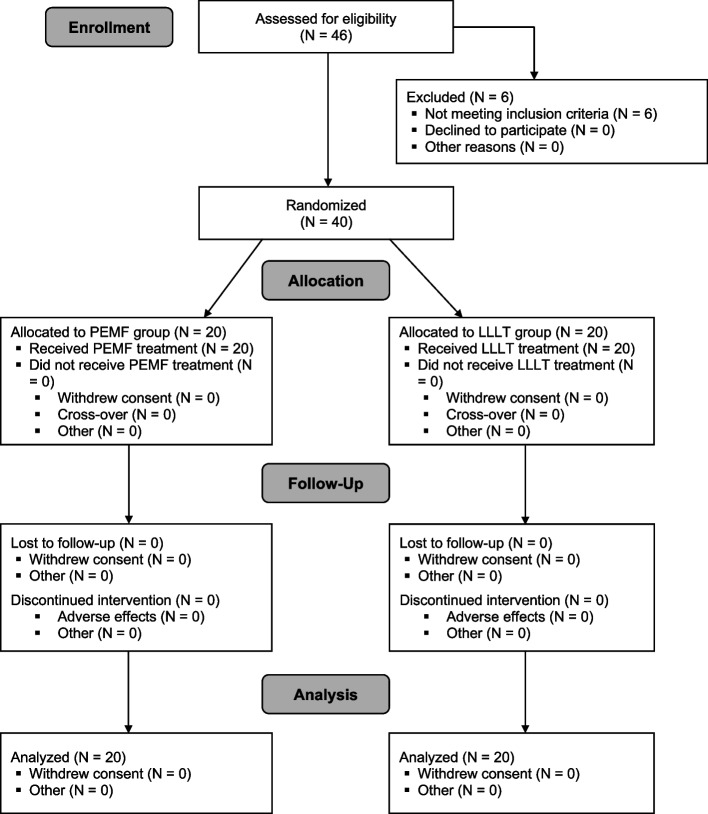
Flow diagram of participants in the study. PEMFT, pulsed electromagnetic field therapy; LLLT, low-level laser therapy

A total of 46 participants were recruited between May 15, 2021 and September 15, 2021. Following recruitment, 40 of the 46 participants met the inclusion criteria and were randomly allocated to one of the two groups (Fig. 1). Six participants were excluded due to the following reasons: previous TKR in the contralateral leg (2 participants), BMI > 40 (2 participants), previous history of lower limb injury in the involved leg within the past month (1 participant), and grade 4 KOA (1 participant). All participants completed six sessions of treatment, with no dropouts. No adverse events were recorded in both treatment groups.

The baseline characteristics of the study participants are detailed in Table 1. The study included 12 men and 28 women, and the mean age of the participants in the PEMFT and LLLT groups were 62.7 and 63.0 years, respectively. The average duration of pain in the PEMFT and LLLT groups was 26.5 and 24.8 months, respectively. Majority of the participants had grade 3 KOA (70%). No statistically significant differences were observed between the two treatment groups in all baseline characteristics.

**Table 1:** Baseline characteristics of patients by group (mean, standard deviation/number, percentage, and *p* value)

Characteristics	PEMFT (***n*** = 20)	LLLT (***n*** = 20)	***P*** value
Age (years, mean ± SD)	62.7 ± 6.6	63.0 ± 6.2	0.95
Sex (n, %):			0.49
Male	7 (35.0)	5 (25.0)	
Female	13 (65.0)	15 (75.0)	
BMI (kg/m^2^, mean ± SD)	31.1 ± 3.3	30.8 ± 3.7	0.84
Pain onset (months, mean ± SD)	26.5 ± 7.5	24.8 ± 7.4	0.42
KL Grade (n, %):			0.49
Grade 2	5 (25.0)	7 (35.0)	
Grade 3	13 (65.0)	15 (75.0)	
Involved side (n, %):			1.0
Right	11 (55.0)	11 (55.0)	
Left	9 (45.0)	9 (45.0)	

The level of significance was set at *p* < 0.05

*PEMFT* Pulsed electromagnetic field, *LLLT* Low-level laser therapy, *SD* Standard deviation, *n* Number, *BMI* Body mass index, *KL* Kellgren–Lawrence classification scale

**Fig. 2:**
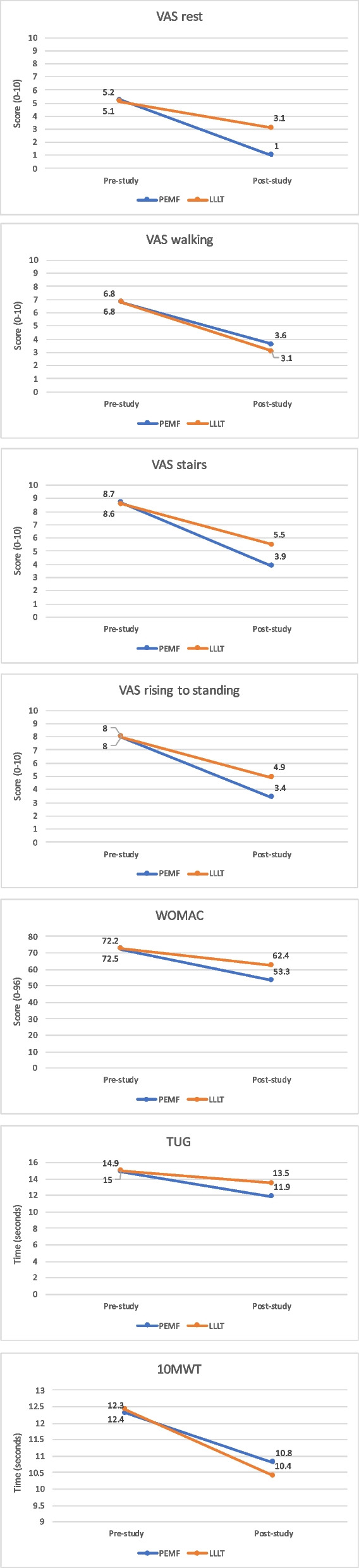
Comparison of the mean delta values of all outcome measures between the two groups. PEMFT, pulse electromagnetic field; LLLT, low-level laser therapy; VAS, visual analog scale; WOMAC, Western Ontario and McMaster Universities Osteoarthritis index; TUG, timed up-and-go test; 10 MW, 10-m walk test

Details of the results of the outcome measures pre/post-intervention and their within-group differences (delta) as well as the results of the comparative analysis of the two groups (*p* values and effect size) are presented in Table 2 and Fig. 2.

**Table 2:** Scores of outcome measures pre/post-intervention and their within-group differences (mean, standard and median) and the results of the comparative analysis of the two groups (*p* values and effect size)

Outcome measure	Measurement time	PEMFT (*n* = 20) Mean ± SD, median	Time effect (*p* value)/effect size	LLLT (*n* = 20) Mean ± SD, median	Time effect (*p* value)/effect size	Group effect (*p* value)/effect size	Group × Time effect (*p* value)/effect size
VAS:							
VAS rest (0-10)	Pre	5.2 ± 1.1, 5.0	< 0.0001 0.50	5.1 ± 1.1, 5.0	< 0.0001 0.50	0.85 0.03	
	Post	1.0 ± 0.9, 1.0		3.1 ± 0.8, 3.0		< 0.0001 0.76	
	Delta	−4.2 ± 1.2, − 4.0		−2.1 ± 0.8, − 2.0			< 0.0001┼ 0.72
VAS walking (0-10)	Pre	6.8 ± 1.0, 7.0	< 0.0001 0.50	6.8 ± 1.1, 7.0	< 0.0001 0.50	0.84 0.03	
	Post	3.6 ± 1.1, 3.5		3.1 ± 0.9, 3.0		0.14 0.2	
	Delta	−3.2 ± 1.0, − 3.0		−3.7 ± 1.1, − 4.0			0.06 0.30
VAS rising to standing (0-10)	Pre	8.0 ± 1.0, 8.0	< 0.0001 0.50	8.0 ± 0.8, 8.0	< 0.0001 0.50	0.8291 0.03	
	Post	3.4 ± 1.0, 3.0		4.9 ± 0.8, 5.0		< 0.0001 0.66	
	Delta	−4.7 ± 1.2, − 5.0		− 3.2 ± 0.9, − 3.0			0.0002┼ 0.58
VAS stairs (0-10)	Pre	8.7 ± 0.6, 9.0	< 0.0001 0.50	8.6 ± 0.5, 9.0	< 0.0001 0.50	0.61/0.10	
	Post	3.9 ± 0.9, 4.0		5.5 ± 1.1, 5.0		< 0.0001 0.63	
	Delta	−3.1 ± 1.0, −5.0		−3.2 ± 1.2, − 3.0			0.0002┼ 0.59
WOMAC:							
Total score (0-96)	Pre	72.2 ± 7.2, 74.5	< 0.0001 0.50	72.5 ± 9.3, 75.5	< 0.0001 0.5	0.63/0.08	
	Post	53.3 ± 9.0, 52.5		62.4 ± 8.7,63.0		0.004 0.45	
	Delta	−19.0 ± 8.0, 19.0		−10.0 ± 3.3, 9.5			0.0002┼ 0.58
Pain subscale (0-20)	Before	16.1 ± 1.9, 16.0	< 0.0001 0.50	15.1 ± 2.0, 15.5	< 0.0001 0.50	0.1390 0.23	
	After	8.4 ± 1.9, 8.0		10.7 ± 1.8, 11.0		0.0009 0.52	
	Delta	−7.7 ± 2.3, −8.0		−4.5 ± 1.3, − 4.0			< 0.0001┼ 0.66
Stuffiness subscale (0-8)	Pre	4.5 ± 1.6, 4.0	0.0013 0.26	3.2 ± 1.8, 3.0	0.0002 0.35	0.02/ 0.36	
	Post	3.3 ± 1.8, 3.0		2.3 ± 1.3, 2.0		0.11 0.25	
	Delta	−1.2 ± 1.2, − 1.0		−0.9 ± 0.8, − 1.0			0.51 0.11
Physical function subscale (0-68)	Pre	51.7 ± 5.5, 51.0	< 0.0001 0.50	54.2 ± 7.2, 56.5	< 0.0001 0.41	0.14 0.23	
	Post	41.6 ± 7.1, 42.0		49.5 ± 7.0, 51.5		0.0015 0.50	
	Delta	−10.1 ± 6.3, − 10.0		−4.7 ± 2.9, − 4.0			0.001┼ 0.52
TUG (seconds)	Pre	14.9 ± 1.3, 15.0	< 0.0001 0.50	15.0 ± 2.0, 15.0	< 0.0001 0.43	0.8907 0.14	
	Post	11.9 ± 1.4,12.0		13.5 ± 1.9, 13.5		0.0068 0.43	
	Delta	−1.6 ± 1.2, −3.0		−1.5 ± 0.9, − 1.5			0.0003┼ 0.57
10 MW (seconds)	Pre	12.3 ± 1.0, 12.0	< 0.0001 0.50	12.4 ± 1.3, 13.0	< 0.0001 0.50	0.6128 0.08	
	Post	10.8 ± 1.2, 11.0		10.4 ± 1.2, 10.0		0.0854 0.27	
	Delta	−1.5 ± 0.9, − 1.5		−2.1 ± 1.2, − 2.0			0.0854 0.27

The level of significance was set at *p* < 0.05

*PEMFT* Pulse electromagnetic field therapy, *LLLT* Low-level laser therapy, *SD* Standard deviation, *Pre* Pre-intervention, *Post* Post-intervention, *VAS* Visual analog scale, *WOMAC* Western Ontario and McMaster Universities Osteoarthritis index, *TUG* Timed up-and-go test, *10 MW* 10-m walk test

┼ - statistically significant

### Pain intensity

Pain intensity in all four activities improved significantly in both treatment groups and had a high effect size (Table 2). However, the difference between the values ​​before and after treatment (delta) was found to be significantly greater, with a high effect size, in three out of the four activities (resting, standing, and climbing the stairs) in the PEMFT group, suggesting that PEMFT is more effective in relieving pain than LLLT. The same results were found in the pain subscale of the WOMAC, with improvement in both treatment groups and significantly greater improvement in the PEMFT group with a large effect size.

### WOMAC score

Both groups showed significant improvement in the total WOMAC scores following the interventions, with greater improvement in the PEMFT group and a large effect size.

### Physical function

As measured by the physical function subscale of the WOMAC and the TUG test, physical function improved significantly in both groups following the interventions; however, there was significantly greater improvement in the PEMFT group than in the LLLT group, with a large effect size.

Although the results of the 10 MW test improved in both groups with a large effect size following the interventions, there was no significant difference between the groups.

## Discussion

This study was the first to compare the effects of PEMFT and LLLT on pain and physical function of participants with primary KOA. Our results indicate that both treatment modalities were similarly effective in reducing pain while walking and improving walking velocity. However, PEMFT was more effective in reducing pain at rest, when standing from a sitting position, and when climbing the stairs. Furthermore, PEMFT had a greater impact on the WOMAC scores and TUG test results. The results of the current study are in accordance with those of previous studies regarding PEMFT, which demonstrated positive effects on pain and physical function [24, 39, 42, 44, 45, 47–50]. Meanwhile, the results of the present study regarding the effects of LLLT on pain and physical function are also supported by the results of previous studies. To the best of our knowledge, only the study of Abdelaal et al. compared these two modalities, with their results indicating that PEMFT was more effective than LLLT in increasing bone mineral density in participants with primary osteoporosis [68]. Moreover, Abdelaal et al. [68] suggested that the advantage of PEMFT is its ability to increase blood supply, resulting in the acceleration of bone organization by osteoblasts [69]. Furthermore, Abdelaal et al. [68] also suggested that PEMFT may not only enhance enzyme-based processes and stimulate growth factors involved in cellular repair and bone formation [70] but may also induce anti-inflammatory effects [68]. While these suggested processes may have affected our results on pain and physical function, our study cannot substantiate these hypotheses; hence, further research is necessary. Notably, however, PEMFT has two technical advantages over LLLT: it does not require special precautions (i.e., dark eyeglasses) during use and it does not require the presence of a caregiver throughout the treatment.

The great variability in the parameters of LLLT and PEMFT used in previous studies is a significant issue that has been consistently raised in the literature, causing difficulties in generalizing conclusions and inability to recommend the optimal parameters of either treatments [22, 24, 28, 29, 33, 35, 36, 47]. This issue is also reflected in the present study wherein the parameters used for PEMFT and LLLT were based on the manufacturer’s recommendations. Thus, the PEMFT parameters in the current study differed from those in the study of Abdelaal et al. [68].

The minimal clinically important difference (MCID) was defined by Jaeschke et al. as “the smallest difference in score in the domain of interest, which participants perceive as beneficial and which would mandate a change in the patient’s management” [71]. The MCID of pain intensity, as measured by the VAS scale, depends on the baseline pain score [14, 72], with higher MCID values expected when baseline pain is high [73, 74]. Tubach et al. [74] categorized the severity of KOA pain on the 10-cm VAS scale as low (3.0–4.9), middle (5.0–6.5), and high (> 6.5). Thus, the MCIDs of the VAS score are 1.1, 2.7, and 3.7 units for the lowest, middle, and highest categories, respectively [74]. In the current study, the subjects’ pain level at rest in both groups prior to treatment was in the midrange, with VAS scores of 5.2 and 5.1 for the PEMFT and LLLT groups, respectively. While the treatments significantly reduced pain at rest, the reduction reached the MCID only in the PEMFT group (≥2.7 units). Unlike pain at rest, baseline pain intensity when standing from a sitting position and when climbing the stairs was high in both groups. However, similar to pain at rest, the treatments significantly reduced pain in both groups, but the reduction in only the PEMFT group reached the MCID (≥3.7 units).

To assess physical function, our study included a self-reported questionnaire (WOMAC) and two objective tests (TUG and 10 MW). The MCID of WOMAC is also linked to the baseline scores, with an MCID of 18.11–22.5 for high WOMAC scores [74]. The baseline WOMAC scores of the PEMFT and LLLT groups were of 72.2 and 72.5, respectively, which are considered to be high (> 51.5) [74]. The current results showed that the WOMAC scores significantly decreased after the treatments; however, only the post-intervention WOMAC score in the PEMFT group reached MCID (19 vs. 9.5, PEMFT vs. LLLT). The baseline TUG parameters in both groups were typical of participants with grades 2–3 KOA [62]. The delta in the TUG score in both treatment groups (PEMFT, 3 s; LLLT, 1.5 s) reached the MCID, which was reported to range between ≥0.8 and 1.4 s depending on the study [75]. We were unable to find details regarding the MCID for the 10 MW test; however, results of the 10 MW test following treatment were not different between the two groups.

This study has certain limitations. First, we did not summarize the patients’ medications prior to performing the interventions and did not limit their use during the intervention period; this may have potentially affected the results. Furthermore, since the study included only individuals with grades 2–3 KOA, the results of the current study cannot be generalized to individuals whose initial KOA grade is < 2 or > 4. Finally, long-term follow-up was not performed; thus, the long-term effects of the interventions could not be determined.

## Conclusions

Both PEMFT and LLLT were effective in reducing pain and enhancing physical function in participants with grades 2–3 primary KOA. PEMFT was more effective than LLLT in improving pain at rest, when standing from a sitting position, and when climbing the stairs. PEMFT was also more effective in improving physical function as reflected by better performances in the TUG test and better scores in the self-reported questionnaire pertaining to physical function. The reduction of pain at rest, when standing from a sitting position, and when climbing the stairs as well as the improvement in the WOMAC scores reached the MCID only in the PEMFT group. Meanwhile, the improvement in the TUG test reached the MCID in both treatment groups. Further research is required to determine and compare the long-term effectiveness of both modalities in treating different grades of KOA. Furthermore, the optimal treatment protocol has yet to be standardized.

## Supplementary Information

**Additional file 1.**

## Data Availability

The datasets used and/or analyzed during the current study available from the corresponding author on reasonable request.

## Abbreviations

BMI: Body mass index
KL: Kellgren–Lawrence
KOA: Knee osteoarthritis
LLLT: Low-level laser therapy
MCID: Minimal clinically important difference
PEMFT: Pulsed electromagnetic field therapy
SD: Standard deviation
TUG: Timed up-and-go test
VAS: Visual analog scale
WOMAC: Western Ontario and McMaster Universities Osteoarthritis Index
10 MW: 10-m walk
