# Supplementary material for: Laser therapy versus pulsed electromagnetic field therapy as treatment modalities for early knee osteoarthritis: a randomized controlled trial
Source: BMC Geriatr. 2023 Mar 16;23:144. doi: 10.1186/s12877-022-03568-5 (PMC10018856; doi:10.1186/s12877-022-03568-5)

Additional file 1

Low level laser therapy (LLLT) device and the points of application


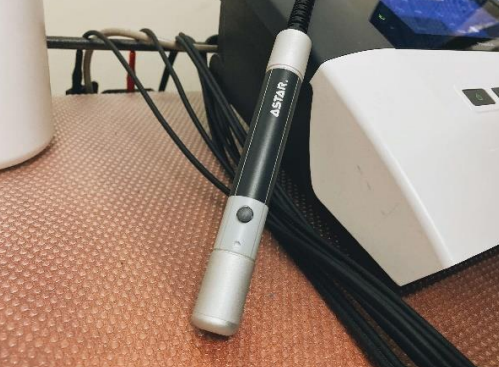

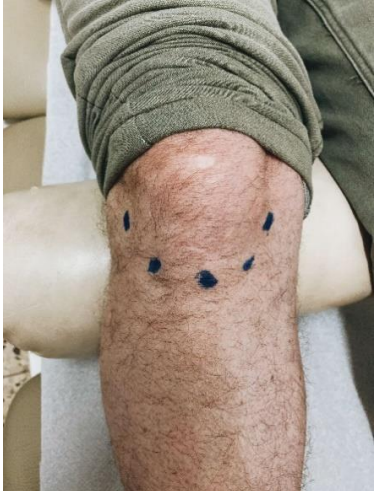


Placement of the pulsed electromagnetic field therapy (PEMFT) device


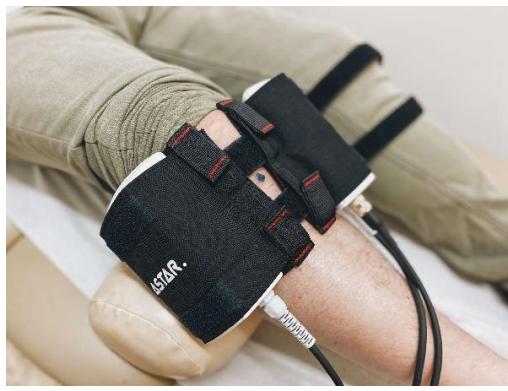

Supplement: Supplementary file 1 — Additional file 1. [file 12877_2022_3568_MOESM1_ESM.docx]
